# Minding the gap: identifying values to enable public and patient involvement at the pre-commencement stage of research projects

**DOI:** 10.1186/s40900-020-00220-7

**Published:** 2020-08-03

**Authors:** Éidín Ní Shé, Jennifer Cassidy, Carmel Davies, Aoife De Brún, Sarah Donnelly, Emma Dorris, Nikki Dunne, Karen Egan, Michel Foley, Mary Galvin, Mary Harkin, Martha Killilea, Thilo Kroll, Vanessa Lacey, Veronica Lambert, Sarah McLoughlin, Derick Mitchell, Edel Murphy, Purity Mwendwa, Emma Nicholson, Deirdre O’Donnell, Laura O’Philbin

**Affiliations:** 1 School of Nursing, Midwifery and Health Systems, University College Dublin, Belfield, Dublin 4, Ireland; 2 Irish Research Council, Dublin, Ireland; 3 UCD School of Social Policy, Social Work and Social Justice, Dublin, Ireland; 4 School of Medicine, University College Dublin, Dublin, Ireland; 5 Family Carers Ireland, Dublin, Ireland; 6 Expert by Experience, Dublin, Ireland; 7 PPI Ignite, Trinity College Dublin, Dublin, Ireland; 8 Design Innovation, Maynooth University, Kildare, Ireland; 9 Age and Opportunity, Dublin, Ireland; 10 PPI Ignite, National University of Ireland Galway, Dublin, Ireland; 11 Transgender Equality Network Ireland, Dublin, Ireland; 12 DCU School of Nursing, Psychotherapy and Community Health, Dublin, Ireland; 13 Irish Platform for Patient Organisations, Science and Industry, Dublin, Ireland; 14 The Alzheimer Society of Ireland, Dublin, Ireland

**Keywords:** Public and patient involvement, Co-design, Pre-commencement stage, Values

## Abstract

**Background:**

The University College Dublin (UCD) Public and Patient Invovlement (PPI) ignite program is focused on embedding PPI in health and social care related research, education and training, professional practice and administration. During a PPI knowledge sharing event challenges were noted during the pre-commencement stage of research projects. This stage includes the time before a research projects/partnership starts or when funding is being applied for. As a response, we agreed there was a need to spend time developing a values-based approach to be used from the pre-commencement of PPI projects and partnerships. Values are deeply held ideals that people consider to be important. They are vital in shaping our attitudes and motivating our choices and behaviours.

**Methods:**

Using independent facilitators, we invited a diverse group of participants to a full-day workshop in February. During the workshop, the concept of a values statement and values-based approaches was introduced. The group via a majority consensus, agreed on a core set of values and a shared understanding of them. After the workshop, a draft was shared with participants for further comment and final agreement.

**Results:**

The workshop had 22 people representing experts by experience, PPI charity partners, funders, academics and national PPI Ignite partners. The group via consensus identified four values of respect, openness, reciprocity and flexibility for the pre-commencement stage. A frequently reported experience of PPI partners was that some felt that the pre-commencement activities appeared at times like a performance; an act that had to be completed in order to move to the next stage rather than a genuine interest in a mutually beneficial partnership. Being open and transparent with all invovled that the funding application may not be successful was stressed. Another important feature related to ‘openness’ was the ‘spaces’ and ‘places’ in which meetings between partners could occur in an accessible and equitable way. The issue of ‘space’ is particularly critical for the involvement of seldom heard groups. The benefits of the research are often clear for academics, but for PPI partners, these are often less certain. To achieve reciprocity, academic and PPI partners need to engage in a timely, repeated and transparent dialogue to achieve beneficial outcomes for all stakeholders. Being open to new inputs and differing modes of knowledge and ideas was also stressed. For some, this will require a change in attitudes and behaviours and should result in more collective decision making. Several areas were identified using the four values.

**Conclusions:**

This work via majority consensus identified four values of respect, openness, reciprocity, and flexibility for the pre-commencement stage. These values should be used to support inclusive, effective and collective PPI across all stages of involvement. We hope this work will stimulate further action in this area. In particular, we would welcome the evaluation of these values involving diverse PPI groups.

## Plain English summary

In Ireland, in 2017, two research funders launched the public and patient involvement (PPI) Ignite program to support a culture of PPI in five universities. In University College Dublin (UCD) the focus is on embedding PPI in health and social care related research, education and training, professional practice and administration. During a UCD PPI Ignite knowledge sharing event by the Alzheimer’s Society on the work of their PPI Dementia Research Advisory, some challenges were highlighted during the pre-commencement stage of research projects. This stage includes the time before a research projects/partnership starts or when funding is being applied for. PPI partners had very little power or influence during this stage. Academics questioned if it was possible to be inclusive due to short time frames and a lack of resources.

As a response, we in the UCD PPI Ignite program agreed to spend time thinking about and deciding what our values are at this pre-commencement stage. Values are deeply held beliefs that people consider to be important. They are important as they share our attitudes and can impact how we behave. We in the UCD PPI Ignite program decided to advance our work by inviting a diverse group of 22 participants to a workshop. This was independently facilitated, which we thought was very important to make sure everyone could contribute. The group agreed on four values. These are respect, openness, reciprocity and flexibility for the pre-commencement stage. During our workshop, we defined each value.

Discussions during the workshop found that for some PPI partners some felt that the pre-commencement activities appeared at times like a performance, like a tick box for researchers completed to move to the next stage rather than a genuine interest in a mutually beneficial partnership. Being open and transparent with all during that a funding application may not be successful was stressed. Another important feature related to ‘openness’ was the ‘spaces’ and places in which meetings between partners could occur in an accessible and equitable way. Often universities are not the best spaces. The benefits of the research are often clear for academics, but for PPI partners, these are often less certain. Academic and PPI partners need to engage in ongoing and clear discussions on what are the benefits of the research for all involved. Being open to new inputs and ideas was also stressed. For some, this will require a change in attitudes and behaviours. Several areas were identified on using the four values, including using the values in the second stage of the ignite program starting in 2021. Our four values should be used to support inclusive, effective and collective PPI. We hope this work will inform more work in this area. In particular, we would welcome the evaluation of these values involving diverse PPI groups.

## Background

We now have a significant amount of literature on values, frameworks and guidance on how to evaluate, report, and share lessons learned from undertaking and enabling public and patient involvement (PPI) [1, 2]. One such example is the framework in which *INVOLVE*, who were funded by the National Institute for Health Research to support public involvement in research. They developed six fundamental values for PPI in research [3]. They are respect, support, transparency, responsiveness, and fairness of opportunity and accountability [3]. They have been tested within PPI collaborations [4]. Others approaches have involved the use of reporting tools such as the *GRIPP2* checklist to enhance transparency of the types of PPI undertaken in health and social care research [5–8]. Yet another example is the public involvement impact assessment framework, which guides researchers on how to capture various types of impact from PPI [9, 10]. A recent systematic review identified a total of 65 frameworks for PPI support and evaluation [11]. These were categorised under five themes of power-focused; priority-setting; study-focused; report-focused; and partnership-focused [11]. The review notes that frameworks were context-specific and often did not transfer to other settings. The study concludes that attention should now shift to enable context-specific co-design activities that involve PPI partners. Crucially, these activities should be independently facilitated to enable equitable involvement [11]. Evidence of context-specific initiatives that are focused on learning and collaborating are now emerging within the literature as best practice [12–14].

In 2017, two national research funders in Ireland, the Health Research Board (HRB) and the Irish Research Council (IRC), launched a joint call entitled ‘PPI Ignite’ to support higher education institutions to embed PPI deeply into their organisational culture. Five universities were initially successful in receiving funding (University College Dublin, National University of Ireland Galway, Dublin City University, Trinity College Dublin and the University of Limerick). Since these academic institutions and their PPI partners have been working together to support PPI knowledge sharing nationally and to create a national network of expertise. Each of the PPI Ignite teams has been working to leave a visible ‘footprint’ within their institutions and have been collaborating and networking externally with PPI partners and collaborators. For example, the UCD PPI ignite program has focused on actively embedding PPI in health and social care related research, education and training, professional practice and administration across UCD structures [15]. We recognise that PPI can occur across a spectrum of activities such as advising on consent sheets, inputting on interview questions, and co-designing all elements of a study from idea development to implementation of findings [15]. A significant focus of our work in UCD PPI Ignite has been on overcoming barriers to the involvement of seldom heard voices, which we have defined in previous work [15, 16].

As part of the UCD PPI ignite program, we undertook a rapid realist review to clarify what was needed to involve seldom heard voices in health and a social care research [16]. We found that PPI partners in community and patient organisations were often approached by academics very close to the grant application deadline and were expected to sign off on the research plan without prior involvement. Frequently researchers came to PPI partners for their signature not offering any process to design the research together from the start. PPI partners were often spending significant time to ensure that the project was culturally appropriate for their population group and accessible [16]. Conversely, the work of researchers often extended beyond the scope of the funded project to provide support to their PPI partners. In many instances, it was junior contract staff who undertook this work. They were spending significant amounts of time with PPI partners to build reciprocal relationships and trust. This ‘invisible’ work was typically not acknowledged by universities in terms of career progression [16]. The rapid realist review concluded that radical changes would be required related to communication, development of protocols guiding continuous involvement among partners, funding and clarity on data ownership [16]. Funders are now encouraging that research grant applicants must include PPI partners as co-applicants [16–19]. This requires the early involvement of PPI partners in the joint development of the research study or program [20]. The literature notes that during this process, PPI co-applicants and researchers may pursue different agendas than can lead to misunderstandings and conflict [21, 22]. In the United Kingdom work has led to the development of an ethical framework for researchers at the early research design stage [22]. In Australia, guidelines from the National Health and Medical Research Council states any research involving Australian Aboriginal people’s needs to demonstrate a return for this marginalised group who ‘have the right to define benefits according to their values and priorities’ [23]. A recent reflective piece in the literature has argued that non-Aboriginal and Torres Strait Islander Peoples and communities researchers must be committed to spending the time to learn about a community’s past and future to enable the development of care and trust within a research partnership [24]. This work reinforces the need to ensure involvement before research commences.

A recent UCD PPI Ignite knowledge sharing event amongst university and external PPI partners in October 2019 involved a presentation by Alzheimer’s Society (LO’P) on the work of their *Dementia Research Advisory* (DRA) team. This team includes a group of people living with dementia and carers who are involved in dementia-research as co-researchers. During the session, some challenges were noted in particular during the pre-commencement stage of research projects. This stage includes the time before a research projects/partnership starts or when funding is being applied for. The DRA team had made significant efforts to promote their availability to potential researchers but had received minimal uptake for their input or involvement. More broadly, it was noted that researchers were often approaching PPI partners towards the end of the application process often requesting sign off from PPI co-applicants on a developed application. As a result, the DRA team felt they had very little power or influence to shape funding applications at this pre-commencement stage. The discussion within the group questioned how feasible it was to be inclusive at this pre-commencement stage due to time pressure or how seldom heard voices could be involved when there is often short time frames for submission. Academics attending outlined how they felt curtailed due to a lack of guidance on what type of involvement was appropriate for this stage. Not surprisingly, a lack of resources was highlighted by all at this pre-commencement stage, meaning that the process was ad-hoc and rushed. As a response to these challenges, we agreed there was a need to spend time developing a values-based approach to be used at the pre-commencement of PPI projects. This paper presents a description of the collaborative co-design approach that led to the development of values.

### What are values

**Fig. 1:**
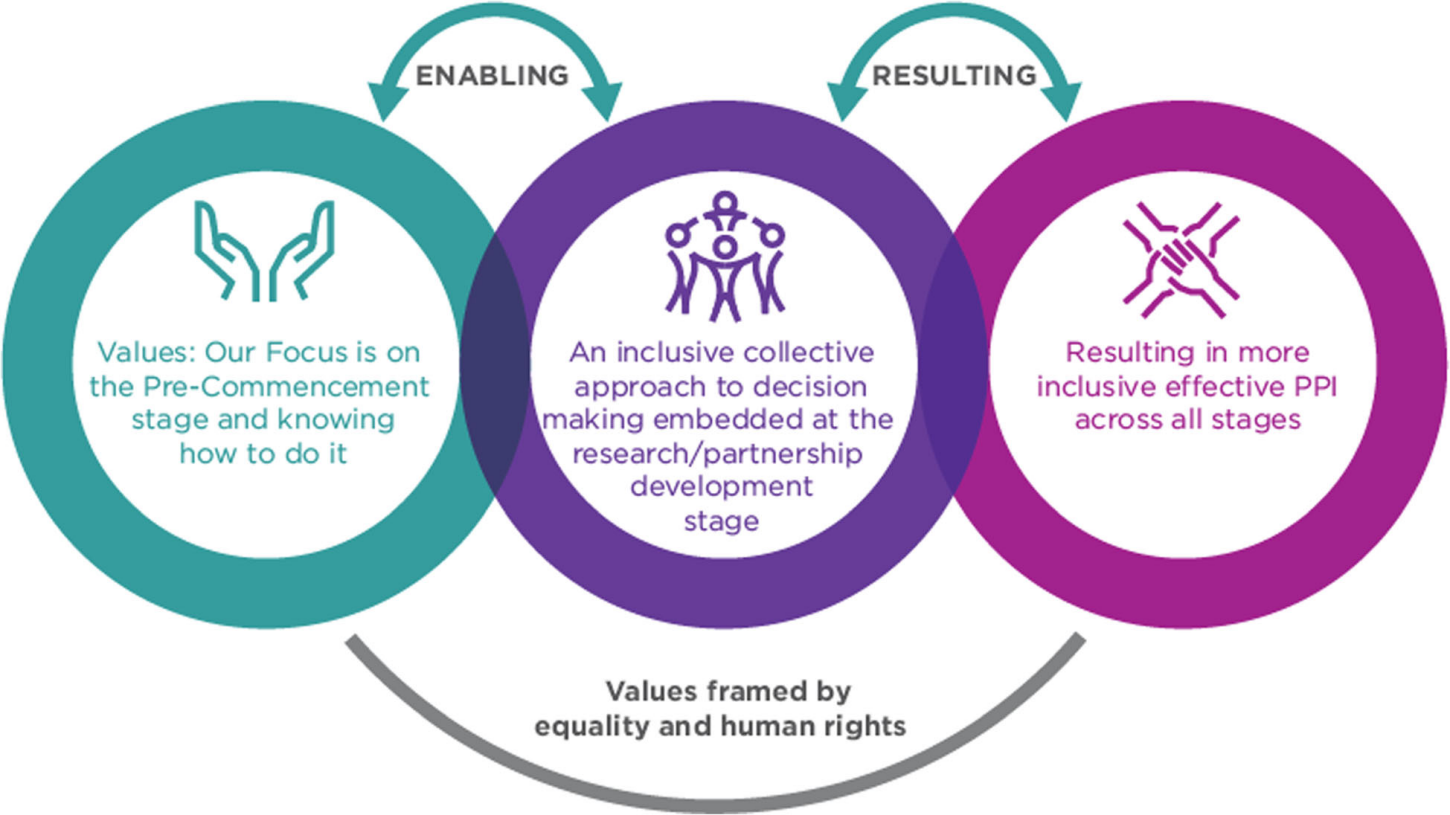
Introducing our focus on using values for PPI from the pre-commencement stage

Values are those deeply held ideals that people consider to be important. They are vital in shaping our attitudes and motivating our choices and behaviours [25]. Making values explicit within PPI is stressed in the literature [26–28]. We wanted to ensure that our values in UCD PPI Ignite were grounded in an understanding of equality and human rights and continued our focus on seldom heard groups. From our initial scoping of the literature and discussions with a diverse cohort of PPI partners, it was agreed that we should focus on the pre-commencement stage. The rationale for the workshop came from the observation that a values-led approach would be fundamental for more inclusive, collective and effective PPI across all stages of involvement (see Fig. 1).

To clarify our thinking and to ensure all perspectives were heard, we aimed to advance our work by inviting a diverse group of participants to a workshop in February 2020 who are involed in PPI in Ireland to agree our vaules. This paper outlines our approach, the agreed values that were reached via consensus and key points from the discussion on them.

## Method

### Independent facilitators

We felt it was essential to have our event independently facilitated as advised in the literature to ensure inclusion of all voices [11]. We approached *Values Lab* to facilitate the workshop. *Values Lab* who are the main organisation in Ireland that supports organisations and networks to enhance their effectiveness by identifying, engaging, and giving expression to their core values [29]. They have a specific focus on engaging and embedding values that motivate the promotion of equality, the prevention of discrimination, and respect for and the protection and fulfilment of human rights. Following consultations with them, it was agreed the work would be completed over three phases.

#### Phase 1: during the workshop

Two independent facilitators introduced the concept of a values statement and values-based approaches to the group. The aim by the end of the dat was for the group to agreed by a majority consensus a core set of values to ensure best practice from the pre-commencement stage of PPI partnership/research development and developed a shared understanding for each value. The group worked in smaller-groups of 4/5 people initially brainstorming on values within groups. Throughout the day the facilitators brought the group together to feedback the group discussion, discuss the values amongst the group fine links and refine them via a majority consensus.

For each agreed value the group in the afternoon session worked on generating outputs to explain them:

- statements of a priority process for each value (what each value suggests regarding to our approach to PPI at the pre-commencement stage); and
- statements of how the value could be practiceed for each value (what each value indicates regarding behaviours relating to individuals and groups when involving the public and patients and)

Finally discussion at the end of the workshop explored how to deploy the developed values statement to full effect.

#### Phase 2: after the workshop

The facilitators drafted the final agreed version of the values statement from the material generated. This draft was circulated by the lead author (ÉNS) to all workshop participants. Following a two week review period any additional feedback was captured before a final draft was developed.

#### Phase 3: finalise work

A final report summary laying out the agreed values was shared with the group.

### Location of the workshop

We agreed it was important that our workshop would take place in a neutral and accessible location for those attending. We wanted to ensure, in particular, that it was away from the university space. Following a review of accessible areas available, we were able to host the workshop at the Irish Human Rights Equality Commission. The commission provides a space for free for community-involved events.

### Summary of participants

All participants who attended are actively involved in the PPI Ignite programs focused on capacity building across Ireland. An invitation to the workshop was extended to all UCD PPI Ignite academic champions, external PPI partners and to the other national univeristy PPI Ignite partners and relevant funders. A total of 22 people attended the workshop and contributed at all stages of the method outlined. All participants are named authors of this paper. Table 1 provides a summary of their background. It should be noted that many attending wear varying hats such as being active patients whilst also working as academics or in charities, some represent a seldom heard group but they also work for a non-government organisation.

**Table 1:** Summary of attendees

Name	Background & Representing
Éidín Ní Shé	Academic & UCD PPI Ignite
Laura O’Philbin	Alzheimer’s Society & Dementia Research Advisory Convenor
Sarah Mcloughlin	PPI Expert by Experience
Deirdre O’Donnell	Academic & UCD PPI Ignite Champion
Michael Foley	Trinity College Dublin PPI Ignite
Nikki Dunne	Family Carer’s Ireland & UCD PPI Ignite Partner Champion
Thilo Kroll	Academic, UCD PPI Ignite
Jennifer Cassidy	PPI Ignite Co-Funder Irish Research Council
Carmel Davies	Academic & UCD PPI Ignite Champion
Karen Egan	PPI Expert by Experience
Emma Dorris	Academic, UCD School of Medicine
Emma Nicholson	Academic, UCD School of Nursing, Midwifery and Health Systems
Sarah Donnelly	Academic & UCD PPI Ignite Champion
Purity Mwendwa	Academic, UCD School of Nursing, Midwifery and Health Systems
Edel Murphy	PPI Ignite @ NUI Galway
Derick Mitchell	Irish Platform for Patient Organisations, Sciences and Industry & UCD PPI Ignite Partner Champion
Aoife de Brún	Academic, UCD School of Nursing, Midwifery and Health Systems
Vanessa Lacey	Transgender Equality Network Ireland
Mary Harkin	Age & Opportunity
Veronica Lambert	Academic & DCU PPI Ignite
Martha Killilea	PPI Ignite @ NUI Galway
Mary Galvin	Academic, Maynooth University

## Results

**Fig. 2:**
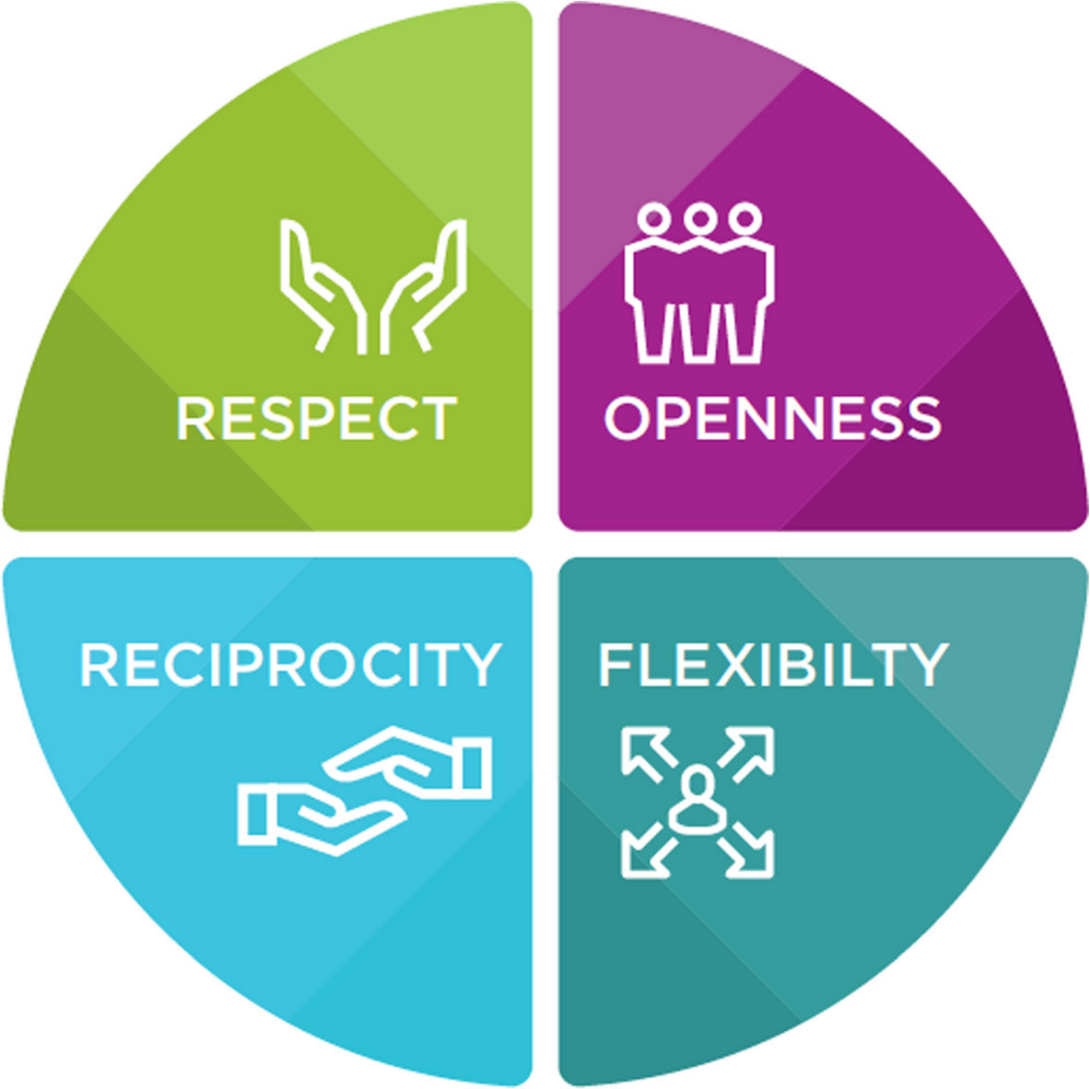
Our agreed values

Group discussion and majority consensus identified four values of respect, openness, reciprocity and flexibility were agreed for the pre-commencement stage (Fig. 2).

**Fig. 3:**
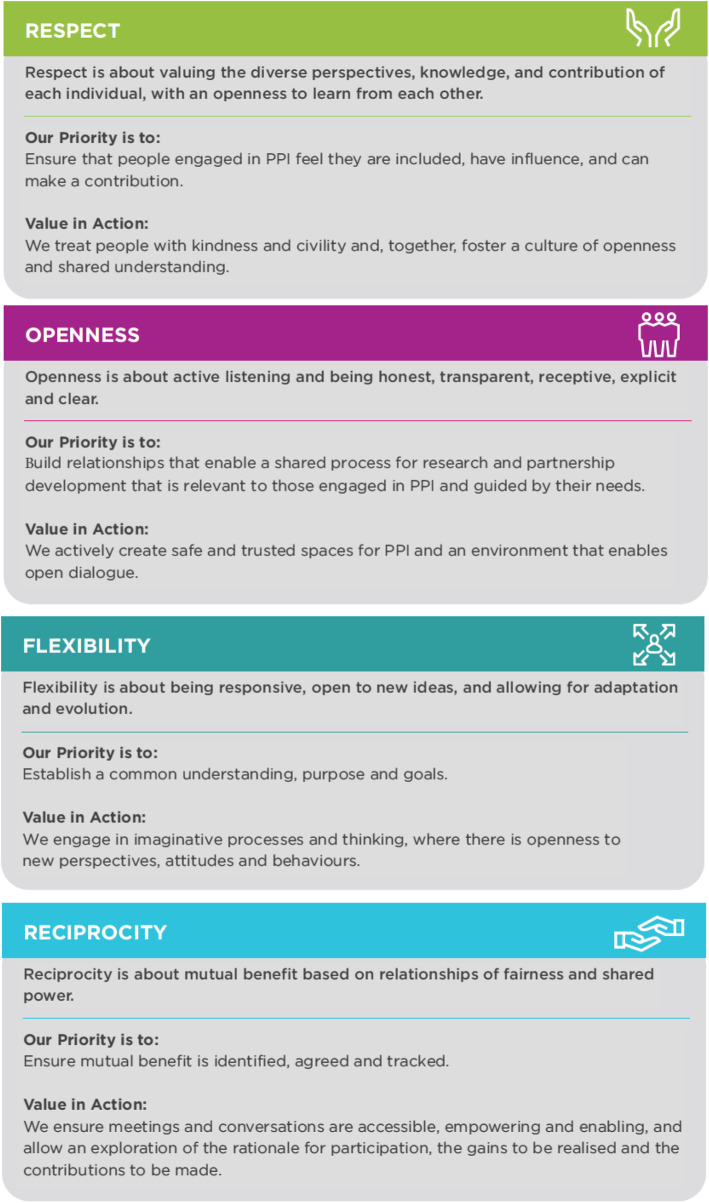
Values Explained

Via majority consensus each of the four agreed values were worked through by the group to agree a priority process and an example of the value in action (Fig. 3).

### Consensus on the agreed values

The two external facilitators systematically guided the group to reach consensus on our core values. The value of ‘respect’ was seen as very important by the group. It is fundamental to the engagement between PPI partners so they can develop a shared understanding of the purpose and direction of their research partnership. From the workshop a frequently reported experience of non-governmental organisations (NGO) partners was that researchers had approached them to discuss research ideas at the pre-commencement stage, which took up a significant amount time. Often the researchers would come with a set research focus and question. The NGO member would spend a considerable amount of their time explaining what their research gaps were and how they were different from the set research focus. This input was frequently overlooked, and no change would occur to the research question. From the NGO perspective, they felt this was a ‘tick box’ exercise where they felt researchers were going through the motions so they could include the meeting into their grant application.

The value of ‘openness’ aligned with the value of ‘respect’ and reinforced the need for a shared process of active listening, ensuring all voices are heard. Openness also requires a critical reflection among stakeholders who holds most power in the relationship. Typically, most power lies with senior researchers in the university, and partners should find ways to mitigate this imbalance. Making sure communication was honest and clear was outlined as important within this value. In particular, being transparent and clear as to the expectations that all partners have. The group discussion revealed that some felt that the pre-commencement activities appeared at times like a performance, an act that had to be completed in order to move to the next stage rather than genuine interest in a mutually beneficial partnership. It was something that had to be carried out until a grant application was submitted. This resulted in overpromising. If funding was received there was often a push back on resources or guarantees, resulting in reduced PPI involvement. Being open and transparent with all during this stage that a funding application may not been successful was also stressed. Another important feature related to ‘openness’ was the ‘spaces’ and places in which meetings between partners could occur in an accessible and equitable way. The issue of ‘space’ is particularly critical for the involvement of seldom heard groups. All interactions should occur in safe and accessible environments. Having these spaces would ensure all partners felt comfortable in speaking up. Universities may not be the best spaces for these initial interactions. Capturing these mutual benefits was also stressed for this value. Keeping a record of activities and how things can change was suggested.

A lot of discussion during the day focused on the issue of language. Specifically, the group felt that it is important that words are used that are understood by everyone. Language should not impose another barrier or generate inequities in terms of who can be meaningfully involved and who cannot. For example, for the value ‘reciprocity’ initially the phrase ‘give and take’ was suggested and discussed within groups at the workshop. Following reflection, the consensus was that the words ‘give and take’ had the potential to reinforce power relations. On the other hand the chosen term ‘reciprocity’ may be very difficult to understand. The group eventually agreed on ‘reciprocity’ as a core value but demanded that a clear, jargon-free description of the term should be included. Basically, ‘reciprocity’ is about the sharing of power that ensures a ‘win-win’ partnership. For example, the benefits of the research are often clear for academics as research outputs will advance their work and careers. For PPI partners, on the other hand, the benefits are often less certain and evident. In order to achieve reciprocity, academic and PPI partners need to engage in a timely, repeated and transparent dialogue to achieve beneficial outcomes for all stakeholders. ‘Reciprocity’ also requires sufficient time for reflection on the merits of getting involved. Thus, a last minute rush to get partners on board is the opposite of ‘reciprocity’.

The final value agreed via consensus was ‘flexibility’. Initially, the discussion focused on merging it with ‘openness’. However when the facilitators helped the group to ‘tease out’ the specific characteristics of ‘flexibility’, and it became clear that it is a distinct value. At its core ‘flexibility’ at the pre-commencement stage is about being open to new inputs and differing modes of knowledge and ideas. It also allows for unconventional ‘out-of-the-box’ thinking and suggests shifts and adaptability should circumstances require these. It brings to bear the complementary knowledge and skills of the entire team. True flexibility also entails that participants do not stick to preconceived notions as to what the focus or outcome would be from the pre-commencement stage. For some, this will require a change in attitudes and behaviours and should result in more collective decision making.

### Using the values

The final part of our workshop shifted the group’s focus on how the four values could be used. Several areas were identified. The first area was aligning these values to national funding schemes. In particular, a second phase of the Ignite program co-funded again by the HRB and the IRC will commence in 2021. A specific focus of the second call will provide seed-funding for research and partnership developing at the pre-commencement stage. The use of these values to capture and evaluate this work could be embedded within these calls was welcomed by all at the workshop. The second area was that the values could be used as a resource in two areas. First in providing capacity building and ongoing training for all PPI stakeholders and secondly within the university teaching curriculum. The values should be communicated widely. In particular to organisations involved in PPI and targeting groups and settings beyond the national ignite programme and via a range of formats. The final identified area was that the values should be used as a benchmark and tool to capture best practice. In particular for reviewing grant applications and peer review research. Clearly, this would require guidance on how to articulate these values in a meaningful way in grant applications. It was also suggested that the values could be used by PPI partners to help them decide whether or not to get involved in the research/ partnership development process. Finally the values could also be used to develop a memorandum of understanding with funders to undertake an evaluation of PPI activity from the pre-commencement stage with all partners invovolved on how to capture impact.

## Discussion

The writing up of our values work has occurred within the unfolding of a unique backdrop. The COVID-19 outbreak has seen a significant shift within Irish funding schemes focused on rapid research and innovation responses [30]. Within the funding call PPI was deemed optional and not seen as essential or suitable within rapid responses. This is disappointing and reinforces that now more than ever PPI is essential and should be values lead. There has also been a considerable amount of discussion online on undertaking PPI during COVID-19 and more specifically about the opportunities and challenges for digital involvement and co-design [31]. Now more than ever PPI involvement should be to the core of our work. We need to ensure that all our responses are values lead from the pre-commencement stage and that they are inclusive of all voices. By identifying and explaining our values, noting what is important about it and giving examples, we believe that we have outlined how they can be used from the pre-commencement stage. We believe that our four values of respect, openness, reciprocity, and flexibility developed by a diverse group of PPI experts by experiences, NGO leaders, funding representative, PPI Ignite leads and academics provides a good foundation to enable inclusive and equitable PPI. It is our aspiration that the four values as outlined here would frame the work of many organisations such as universities, funders and PPI networks and should also serve as a basis for developing long term reciprocal partnerships.

Recent work has argued that there has been a saturation in the literature on principles and values, suggesting focus should shift to implementation and evaluation [32]. Our work responded to the gap in the literature by focusing explicitly on the pre-commencement stage where research is developed, or initial partnerships are explored. We believe from our PPI work that this where there are gaps in the evidence, and it is where we believe that the inequalities of power and agenda-setting are occurring. We are encouraged to observe that the recent PPI evidence has started to shift focus on this pre-commencement stage [16, 33]. There is clear agreement from those who undertake PPI co-production work on the need to move away from “fund and forget models” [16]. Responses to these gaps need to support long-term reciprocal partnerships. However, there are plently of challenges in achieving this. Notably, a major challenge is the lack of funding schemes in enabling equal collaboration at the pre-commencement stage [16, 32, 34]. We recognise that funding for the pre-commencement stage is important, but much work is also needed internally within universities structures to recognise, support, and enable this phase of work [16, 33–36]. Without a focused attention on changing the culture, the status quo will remain. Our focus within the UCD PPI ignite program has been developing alliances across the university towards changing the culture, and developing a community of practice to support understanding on public engagement. This work is ongoing via UCD PPI Ignite, and the key focus is ensuring that the work is recognised within career pathways.

We recognise that undertaking PPI work requires diverse modes of involvement strategies [16, 36, 37]. We have outlined why this work must be values-led from the pre-commencement stage. This will fill the gap and support more inclusive and effective PPI across all stages, methods and approaches of involvement. By identifying areas where our four values could be used, we have direct implementation pathways via stage two of the PPI ignite program for these values.

### Limitations of this work

The values from this work are based on the majority consensus of a small number of participants (*n* = 22). Getting the right type of PPI partners in a room is an ongoing challenge. We included our PPI Ignite partners both internally and externally to UCD to ensure the values can be implemented within the Irish context. It should be stressed that the debate and discussion during the workshop were inclusive and disagreement did emerge around language, what is feasible during the pre-commencement stage due to a lack of resources and time. Consensus would not have been achieved had the session been run by the group without an external facilitators. Having a neutral facilitator was a key enabler for consensus but we recognise the additional cost may not be available to all. Hosting the workshop in a neutral venue was also valuable as it was a space that the majority had not used before and we were fortunate that we did not have to incur a cost to use it something that may not be available to all. The UCD PPI Ignite program was able to support the costs as the work will feed into the second stage of the national PPI Ignite program. Other funders nationally and internationally should look at enabling involvement at the pre-commencement stage. It would be important to conduct values work that is context-specific to explore whether the values are generalisable. We hope this work will stimulate further work in this area. In particular, we would welcome the evaluation of these values.

## Conclusion

This work via collaborative consensus identified four values of respect, openness, reciprocity, and flexibility for the pre-commencement stage that should support more inclusive and effective PPI across all stages of involvement. Our work identified area where these values can be implemented to support inclusive, effective and collective PPI across at stages. A significant success in the workshop was the space created for all participants to contribute and debate the values. This was the role of the independent facilitators and in our view should be an essential requirement to support sense-making and consensus.

## Abbreviations

PPI: Public and Patient Involvement
HRB: Health Research Board
IRC: Irish Research Council
UCD: University College Dublin
DRA: Dementia Research Advisory
NGO: Non-governmental organisation
